# 2,4-D Herbicide-Induced Hepatotoxicity: Unveiling Disrupted Liver Functions and Associated Biomarkers

**DOI:** 10.3390/toxics12010035

**Published:** 2024-01-02

**Authors:** Rafael Xavier Martins, Matheus Carvalho, Maria Eduarda Maia, Bruno Flor, Terezinha Souza, Thiago Lopes Rocha, Luís M. Félix, Davi Farias

**Affiliations:** 1 Post-Graduation Program in Biochemistry, Department of Biochemistry and Molecular Biology, Building 907, Campus Pici, Federal University of Ceará, Fortaleza 60455-970, Brazil; rafaelxavier@alu.ufc.br (R.X.M.); eduardamaia95@gmail.com (M.E.M.); 2 Laboratory for Risk Assessment of Novel Technologies, Department of Molecular Biology, Federal University of Paraiba, João Pessoa 58050-085, Brazil; asfdavsk@gmail.com (M.C.); brunomifla1@gmail.com (B.F.); terezinhamsouza@gmail.com (T.S.); 3 Laboratory of Environmental Biotechnology and Ecotoxicology, Institute of Tropical Pathology and Public Health, Federal University of Goiás, Goiânia 74055-110, Brazil; thiagorochabio20@ufg.br; 4 Centre for the Research and Technology of Agro-Environment and Biological Sciences (CITAB), University of Trás-os-Montes and Alto Douro (UTAD), 5000-801 Vila Real, Portugal; lfelix@utad.pt; 5 Inov4Agro, Institute for Innovation, Capacity Building and Sustainability of Agri-Food Production, University of Trás-os-Montes and Alto Douro (UTAD), 5000-801 Vila Real, Portugal

**Keywords:** emerging contaminant, oxidative stress, water pollution, agrochemical

## Abstract

2,4-dichlorophenoxyacetic acid (2,4-D) is a widely used herbicide worldwide and is frequently found in water samples. This knowledge has prompted studies on its effects on non-target organisms, revealing significant alterations to liver structure and function. In this review, we evaluated the literature on the hepatotoxicity of 2,4-D, focusing on morphological damages, toxicity biomarkers and affected liver functions. Searches were conducted on PubMed, Web of Science and Scopus and 83 articles were selected after curation. Among these studies, 72% used in vivo models and 30% used in vitro models. Additionally, 48% used the active ingredient, and 35% used commercial formulations in exposure experiments. The most affected biomarkers were related to a decrease in antioxidant capacity through alterations in the activities of catalase, superoxide dismutase and the levels of malondialdehyde. Changes in energy metabolism, lipids, liver function, and xenobiotic metabolism were also identified. Furthermore, studies about the effects of 2,4-D in mixtures with other pesticides were found, as well as hepatoprotection trials. The reviewed data indicate the essential role of reduction in antioxidant capacity and oxidative stress in 2,4-D-induced hepatotoxicity. However, the mechanism of action of the herbicide is still not fully understood and further research in this area is necessary.

## 1. Introduction

2,4-Dichlorophenoxyacetic acid (2,4-D) is an herbicide derived from phenoxyacetic acid. It has been widely used in agriculture since 1946 for weed control [1,2]. This chlorinated aromatic hydrocarbon was one of the first synthetic herbicides to enter the market and is currently the main ingredient in over 1500 products available in the market, such as Weedestroy^®^ AM40 and DMA^®^ 4 IVM [3,4].

The 2,4-D herbicide mimics the effects of auxins, hormones that regulate plant growth [5]. Due to its high water solubility, the herbicide is able to reach conducting vessels through plants leaves and roots, thus spreading throughout the plant, causing abnormal tissue growth and other deleterious effects that ultimately lead to plant death [5,6,7,8]. Due to its high efficiency and low-cost, 2,4-D is globally used as a pre- and post-emergence herbicide in crops such as rice, coffee, sugarcane, corn, and soybeans [6,9,10].

Countries with greater agricultural activity exhibit a prominent use of this herbicide. According to the United States Department of Agriculture, the USA, South America, Europe, and Russia are the primary markets and producers of 2,4-D, with their consumption experiencing a significant 40% increase over the last decade [11,12]. In the United States, approximately 600 agricultural and residential products contain 2,4-D as the active ingredient. Furthermore, in 2012, it was the fifth most widely applied herbicide in the agricultural sector of the country [13,14,15]. In Argentina, over 2000 tons of 2,4-D are employed in various crop types, particularly in glyphosate-tolerant corn and soybean cultivation [11,16]. In Brazil, a total of 62,165.70 tons of 2,4-D were sold in 2021, making it the second best-selling agrochemical in the country, a prominent position maintained since 2013 [17]. 2,4-D is also highly consumed in Asian countries. In Thailand, 2,4-D emerged as the most imported herbicide in 2021, totaling 11,781 tons [18]. In China, the production of 2,4-D reached a significant milestone of 40,000 tons in 2010 [19].

Considering that products applied to crops undergo leaching and have access to water bodies, the high level of commercialization of 2,4-D is evident in its frequent detection in surface, groundwater, and drinking water samples [5]. Furthermore, 2,4-D is environmentally persistent, with a half-life in water ranging from 15 to 300 days depending on environmental parameters [20,21,22,23]. Concentrations of 2,4-D detected in aquatic environments usually range from 4 to 24 µg/L [3,24]. In surface water, 2,4-D has been detected at varying concentrations around the world. In Spain, the detection in drinking and surface water ranged from 62 to 207 ng/L, while in the United States it was found to be between 0.1–12 μg/L in urban surface water and sediments [25,26]. Likewise, in Australia, 2,4-D was identified in urban waterways at a concentration of 3.5 ng/L [27]. In northern Iran, 2,4-D was detected in river water at 16.6 μg/L [28]. In Greece, similarly in river water, the herbicide was detected at 1.16 μg/L [29]. Furthermore, values ranging from 359 to 656 µg/L have already been detected in surface water shortly after herbicide spraying in plantation settings [3,23].

In Brazil, according to data obtained by the Ministry of Health through the Water Quality Surveillance Information System for Human Consumption (SISAGUA), 2,4-D was detected in 92% of samples of water used to supply more than 2300 cities in Brazil between 2014 and 2017 [30]. Despite only two detections showing concentrations above the permissible limit in Brazil (30 µg/L), 4270 detections exhibited values exceeding the limit set by the European Union, which adopts a more conservative stance (0.1 µg/L) [30,31]. The concern about the presence of 2,4-D in water lies in its toxic effects on non-target organisms. In terms of these effects, there is a growing number of studies in the literature that highlight its hepatotoxicity in different biological models (e.g., rodents and fish). The effects include liver cellular and tissue damage, the inhibition of hepatic antioxidant enzymes, lipid peroxidation (LPO), and increased seric levels of transaminases [32,33,34].

The liver plays vital functions in vertebrates, including nutrient metabolism and detoxification processes [35]. Hepatic damage can have negative impacts on these processes and lead to the development of several diseases such as fibrosis, cirrhosis, steatosis, and hepatocellular carcinoma [35]. Therefore, the current study aimed to summarize and analyze, from a critical standpoint, the available literature on the hepatotoxicity induced by pure 2,4-D or commercial formulations containing it as the active ingredient. To do so, we addressed markers of toxicity and affected liver functions, as well as biological models, chemical compounds, effects in mixtures, and hepatoprotection assays. Research gaps and recommendations for future studies were also addressed.

## 2. Materials and Methods

The articles used in the literature review were obtained from the PubMed, Web of Science and Scopus databases, and the search covered all papers published until July 2023. Two keyword combinations were used: (i) “2,4-D” and “liver”; and (ii) “2,4-D” and “hepatotoxicity”. The decision to utilize the abbreviation 2,4-D rather than its full nomenclature in the database queries was motivated by the higher volume of located articles and the consistency in outcomes across various keyword combinations. The articles found were curated according to the following inclusion and exclusion criteria:

(i) Inclusion criteria: articles written and published in English; original and experimental articles; articles that used pure 2,4-D or commercial formulations containing it as the active ingredient; articles that used vertebrates or derivatives (e.g., cells, organelles, enzymes) as biological models; and articles that used biological samples derived from hepatic tissue.
(ii) Exclusion criteria: articles that were not written and published in English; articles that studied other chemical compounds but not 2,4-D; articles that did not use vertebrates or derivatives as biological models; review articles; clinical cases, efficacy studies, protocols, technical reports, and studies that did not meet the research aims.

Subsequently, the selected articles were examined for relevant information on the theme of the review. The extracted information included: DOI, year of publication, geographic location of the study identified by the corresponding author’s address, nature of the chemical compound (active ingredient or commercial formulation), biological model used, route of administration/exposure, exposure period, evaluated concentrations, morphological liver damages and toxicity biomarkers.

### Overview

**Figure 1:**
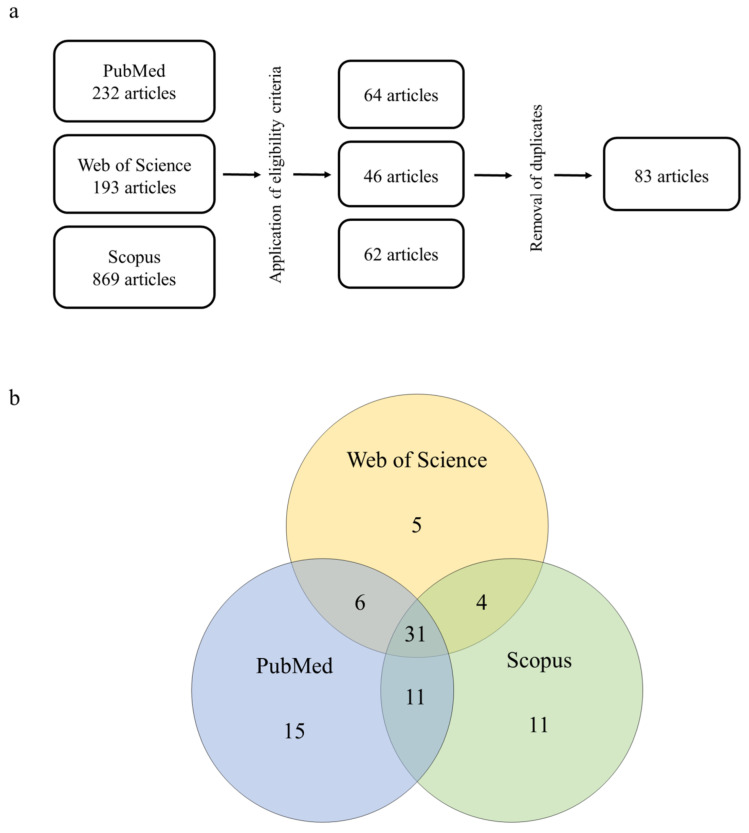
(**a**) Methodological approach to the systematic review of 2,4-D herbicide-induced hepatotoxicity and (**b**) degree of overlap across the databases queried in this study.

The searches in PubMed using the keyword combinations resulted in a total of 232 findings. After applying the inclusion and exclusion criteria, 64 articles remained. Using Web of Science, a total of 193 findings were identified, and 46 articles remained after applying the eligibility criteria. In Scopus, a total of 869 articles were identified, from which 62 were selected. At the end of the curation process and after the removal of duplicates, a total of 83 articles remained, with 31 of them being present in all three databases (Figure 1a,b). These articles are summarized in Table 1 (in vivo studies) and Table 2 (in vitro studies).

The toxicity biomarkers identified were grouped based on their relationship to different aspects of liver physiology [115,116,117]. The categories were: antioxidant metabolism (AM), energetic metabolism (EM), lipid metabolism (LM), liver function (LF), and xenobiotic metabolism (XM). The biomarkers grouped in the LF category are utilized in both clinical diagnosis and research to investigate liver damage and function, including AST, ALT, and ALP [118]. Effects that were not directly related to any specific category were allocated to “not determined” (ND). Markers related to more than one category were placed in both groups (e.g., glutathione S-transferase and glutathione were allocated to both AM and XM). After this classification, it was possible to determine which categories were evaluated by each article in the review and identify the toxicity markers that appeared most frequently in the studies. Additionally, eight studies were identified as containing information on the hepatotoxic effects of 2,4-D in association with other agrochemicals, and eight studies focused on hepatoprotection against damage induced by the herbicide.

## 3. Results and Discussion

### 3.1. Historical Review and Geographical Distribution

**Figure 2:**
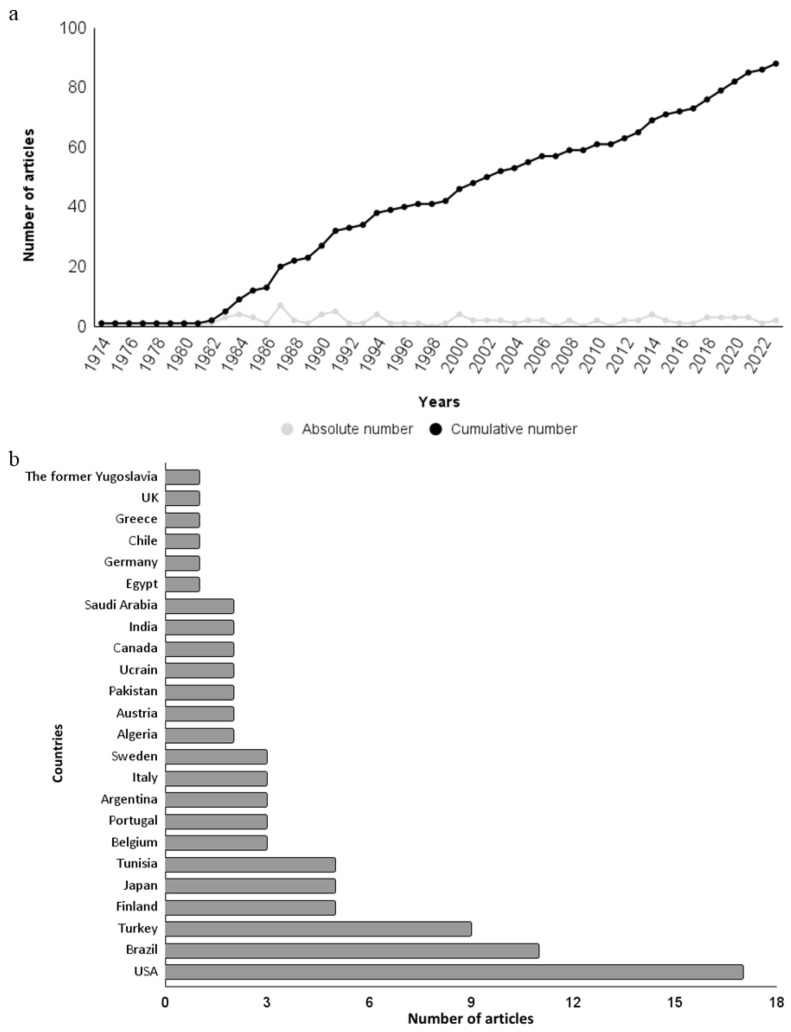
Bibliometric data of articles on the 2,4-D herbicide induced hepatotoxicity. (**a**) Absolute and cumulative number of articles over the years. (**b**) Number of articles per country.

The article published by Olson et al. (1974) was the oldest study on the hepatotoxicity of 2,4-D [112]. Since then, a significant number of studies have been published (Figure 2a). It is noteworthy that approximately 55% (*n* = 46) of the articles in this review were published prior to the year 2000. In part, the high number of older studies can be explained by the fact that 2,4-D was the first synthetic herbicide to be developed (in the 1940s) and it was used worldwide in agriculture, which sparked the interest of research groups in studying its effects on non-target organisms [1]. In addition to its use in agriculture, the fact that 2,4-D is one of the components of Agent Orange, a defoliant widely used during the Vietnam War, may have also contributed to the significant number of studies conducted during that period [3].

The studies identified in the review were conducted by research groups from 23 countries, most notably the USA (*n* = 17; 20%), Brazil (*n* = 11; 13%) and Turkey (*n* = 9; 11%) (Figure 2b). In both countries, agriculture plays a significant economic role, and 2,4-D is widely commercialized [14,17].

### 3.2. Chemical Compounds

**Figure 3:**
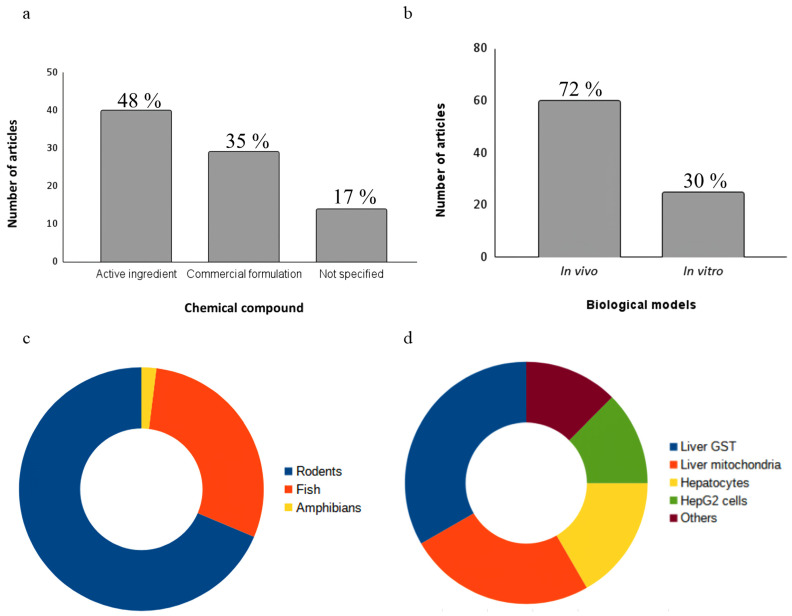
(**a**) Types of 2,4-D formulation used in hepatotoxicity studies (**b**) across in vivo and in vitro models. (**c**) Types of in vivo models used and (**d**) types of in vitro models used.

In the toxicity assays of the selected studies, 2,4-D was used in either its pure form (*n* = 40; 48%) or via a commercial formulation containing it as the active ingredient (*n* = 29; 35%). Articles that did not specify the origin of the substance used in the exposure assays were classified as “not specified” (*n* = 14; 17%) (Figure 3a).

Désormone Lourd (600 g/L), U46D-Fluid (868 g/L), Vesakontuho tasku (500 g/kg), and Tordon 75D^®^ (300g/L 2,4-D + 75 g/L picloram) are examples of 2,4-D-based commercial formulations used in the studies of this review. Commercial formulations are cocktails containing one or more active ingredients and other substances known as inert or adjuvant ingredients (e.g., surfactants, solvents, and preservatives). These substances serve to improve the dissolution, stability, absorption, and pesticidal action of the active ingredient [119]. However, adjuvants can have biological activity and influence the toxicity of the active ingredient [120]. This makes it difficult to compare experimental data since different formulations vary with regard to the composition and concentration of these compounds [119]. Furthermore, among the analyzed studies, only one conducted a comparative analysis of the hepatotoxic effects of the active ingredients of the herbicide (Tordon 75D^®^) and its inert ingredients [103]. Comparative studies are essential for evaluating differences in toxicity among different 2,4-D formulations, including the pure herbicide, and identifying variations in toxic effects due to adjuvants [120]. This highlights the lack of such studies in the literature, which could provide important information for establishing safe limits for these compounds in the environment.

### 3.3. Biological Models

Approximately 72% (*n* = 60) of studies used in vivo biological models, while around 30% (*n* = 25) used in vitro models (Figure 3b). Only the study by Di Paolo et al. (2001) employed both approaches [56]. In vivo models have advantages in hepatotoxicity studies compared to in vitro models because they consider the interactions between different liver cell types, as well as the influence of systemic factors [121]. Revised results showed that further studies using organs-on-chips (OoCs) and body-on-a-chip (BoC) platforms are needed since these methodologies create the environments that recapitulate one or more tissue-specific functions [122].

Regarding the in vivo models, 68% (*n* = 41) used rodents, 27% (*n* = 16) used fish, and 5% (*n* = 3) used amphibians (Figure 3c). Among these, 18 different species were identified, with *Rattus norvegicus* and *Mus musculus* being the notable ones (Table 1). Rodents are widely used in hepatotoxicity studies due to their high morphophysiological, biochemical, and genetic homology with humans, including liver metabolism [123]. Fish, such as *Poecilia vivipara*, *Cyprinus carpio* and *Rhandia quelen*, are organisms with sensitivity to changes in environmental parameters and are directly affected by the presence of agrochemicals in aquatic bodies [124]. Figure 3d depicts in vitro biological models. Among them, the most commonly used were the inibithion assay of isolated hepatic glutathione S-transferase (GST) (*n* = 7, 28%). Isolated liver mitochondria from *R. norvegicus* were also used as a model (*n* = 6, 24%), along with hepatocyte cultures (*n* = 4, 16%) and HepG2 cells (*n* = 4, 16%). The remaining studies utilized liver and chicken embryo and were categorized as “other” (*n* = 2, 8%). More information about the in vitro biological models can be found in Table 2.

### 3.4. Morphological Markers

The liver is composed of different cell types, such as hepatocytes, Kupffer cells, stellate cells, and hepatic sinusoidal endothelial cells [125]. The composition and organization of these cells in the organ vary according to the species, but in general hepatocytes are more abundant and perform many of the hepatic functions [126].

Data collected in this review show that 2,4-D causes various impacts on liver tissue. Macroscopically, exposure to 2,4-D induces hepatomegaly in rodents. Male rats treated with 150 mg/kg of Désormone Lourd (600 g/L 2,4-D) for 4 weeks showed approximately a 43% increase in absolute organ weight compared to the negative control [63]. At the cellular and tissue level, histopathological analyses were predominant in identifying damages in the reviewed articles. Rats treated with 15 mg/kg of Désormone Lourd (600 g/L 2,4-D) for 4 weeks exhibited cellular death, which was indicated by the formation of pyknotic nuclei and focal necrosis [63]. Additionally, 5 mg/kg of the same commercial formulation induced perivascular inflammatory infiltration in the liver of rats, indicating the presence of immune system cells and other components involved in the inflammatory response [32]. In addition to inflammatory effects, dietary exposure to 2,4-D has been previously associated with a high incidence of hepatic steatosis in rats [72]. It is important to emphasize that the mentioned morphological changes are associated with a decrease in hepatic antioxidant function, indicated by the reduced activity of antioxidant enzymes such as superoxide dismutase (SOD), catalase (CAT), glutathione S-transferase (GST), glutathione peroxidase (GPx), and hepatic GSH levels [32,63]. Morphological damage can also contribute to hepatobiliary disorders. Tichati et al. (2020) showed an increase in alkaline phosphatase (ALP) activity and total bilirubin levels, which are used as indicators of biliary flow dysfunction [32]. Furthermore, obstruction of the bile ducts can lead to the accumulation of bile salts in the liver, exposing hepatocytes to toxic concentrations of bile acids and enhancing hepatic injuries [127].

Other histopathological changes frequently observed after exposure to 2,4-D include hepatocyte vacuolization and deterioration of the liver tissue structure. Cattaneo et al. (2008) identified these effects in the liver of *R. quelen* after exposure to 700 mg/L of U46D-Fluid (868 g/L 2,4-D) for 96 h [83]. Similar effects were found in the liver of zebrafish larvae at 120 h post-fertilization (hpf) when exposed to pure 2,4-D (>97%) at 2.5, 5, and 10 mg/L [4]. 

The morphological changes found by Cattaneo et al. (2008) were accompanied by an impairment in liver energetic function, as evidenced by blood glucose alterations and a decrease in hepatic glycogen level [83]. These effects are commonly associated with disruptions in gluconeogenesis and glycogenolysis pathways, which play crucial roles in maintaining blood glucose levels [63,128]. Moreover, an increase in hepatic lactate dehydrogenase (LDH) activity was observed, suggesting enhanced anaerobic glucose metabolism. This could possibly be attributed to tissue damage and reduced oxygen availability [83].

### 3.5. Toxicity Biomarkers

#### 3.5.1. Antioxidant Metabolism

**Figure 4:**
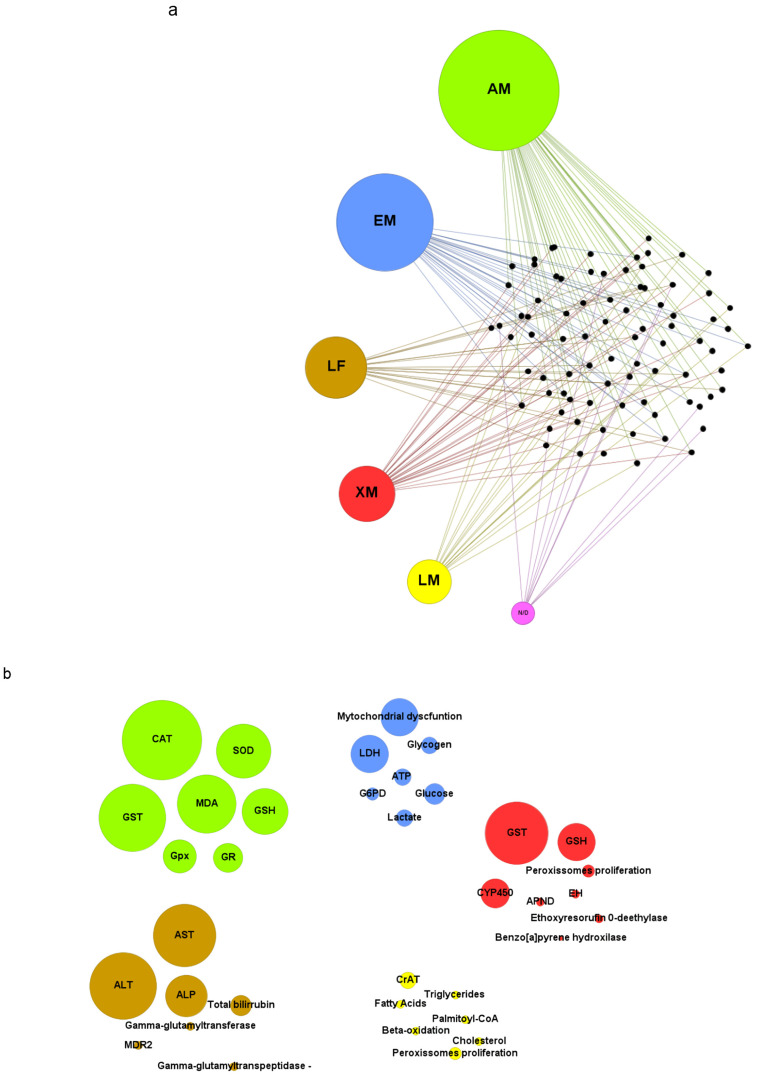
(**a**) Selected articles in the review (small black dots) and the hepatic functions that were affected: antioxidant metabolism (AM), energetic metabolism (EM), liver function (LF), lipid metabolism (LM), xenobiotic metabolism (XM) and not determined (ND). (**b**) The top seven disturbed biochemical markers for each hepatic function. Size circles correspond to the number of occurrences in the articles. Abbreviations: catalase (CAT), superoxide dismutase (SOD), glutathione S-transferase (GST), malondialdeíde (MDA), glutathione peroxidase (GPx), reduced glutathione (GSH), glutathione reductase (GR), lactate dehydrogenase (LDH), glucose 6 phosphate dehydrogenase (G6PD), alanine aminotransferase (ALT), aspartate aminotransferase (AST), alkaline phosphatase (ALP), multidrug resistance protein 2 gene (MDR2), glutathione S-transferase (GST), cytochrome p450 enzymes (CYP450), epoxide hydrolases (EH), amidopyrine N-demethylase (APND), carnitine acetyltransferase (CrAT).

Toxicity biomarkers associated with antioxidant metabolism and oxidative stress were the most frequent, reported in 49% (*n* = 41) of the reviewed articles (Figure 4a). Free radicals, such as reactive oxygen species (ROS) and reactive nitrogen species (RNS), are natural byproducts of cellular aerobic metabolism [129]. However, when the production of free radicals exceeds the antioxidant capacity of the organism and reaches high concentrations, oxidative stress occurs, resulting in oxidative damage to cellular macromolecules, such as proteins, lipids, and nucleic acids [35,130].

The decrease in antioxidant capacity was mainly shown by the reduction in the activity of hepatic antioxidant enzymes, such as superoxide dismutase (SOD), catalase (CAT), glutathione S-transferase (GST), glutathione peroxidase (GPx), and glutathione reductase (GR) [62,64,75]. Among them, CAT (*n* = 19, 46%), GST (*n* = 16, 39%), and SOD (*n* = 13, 31%) were the most frequently reported in the articles that investigated markers related to the antioxidant response. (Figure 4b). SOD is responsible for converting the superoxide radical (O_2_^−^) into a less reactive form, hydrogen peroxide (H_2_O_2_), while CAT decomposes H_2_O_2_ into water and oxygen [131]. GST, GPx, and GR are involved in the regulation and metabolism of glutathione (GSH), a crucial non-enzymatic antioxidant involved in neutralizing free radicals and eliminating endogenous and exogenous toxic compounds from the body [132].

Exposure to 2,4-D also leads to the depletion of GSH levels in the liver, contributing to a decrease in antioxidant capacity [71,119]. This effect was observed in rats receiving doses of 5 mg/kg of Désormone Lourd (600 g/L 2,4-D) for 4 weeks, resulting in a decrease in hepatic GSH levels and the activity of SOD, CAT, GPx, and GST [33]. Toxicity studies conducted with the active ingredient in rats identified similar results, further supporting that this effect is attributed to the active ingredient [71,74].

The decrease in antioxidant response and induction of an oxidative stress state leads to cellular and tissue damage. This is evidenced by the increase in MDA levels, an effect frequently reported in the articles (*n* = 14, 34%) [34,60]. This alteration indicates the oxidative degradation of lipids (LPO) induced by 2,4-D. MDA is a product of lipid peroxidation, especially of polyunsaturated fatty acids, and is considered a common marker of oxidative stress and oxidative damage to lipids and cell membranes [34]. Hepatic cells are particularly vulnerable to oxidative stress from various toxic agents because the liver serves as the primary site for drug metabolism [35]. Furthermore, oxidative stress plays a crucial role in the progression of liver diseases induced by toxic chemical compounds, such as nonalcoholic fatty liver disease (NAFLD) [129]. These data suggest that oxidative stress plays an important role in the progression of 2,4-D-induced hepatotoxicity. 

#### 3.5.2. Energetic Metabolism

The analyzed articles demonstrate that markers associated with energetic metabolism are disrupted following exposure to 2,4-D, as was reported in 36% (*n* = 30) of the studies (Figure 4a). Among them, alterations associated with mitochondrial dysfunction were frequent (*n* = 9, 30%) (Figure 4b). 2,4-D can inhibit the activity of mitochondrial enzymes and cause a depletion in cellular ATP levels, compromising the availability of energy required for proper functioning of the hepatic cells [102,104]. Isolated liver mitochondria treated with 600 µM of 2,4-D showed inhibition of complex III (cytochrome c reductase) [104]. Additionally, complex I (NADH cytochrome c reductase) was also inhibited in liver mitochondria exposed to 13.2 nmol/mg of the herbicide Tordon 2,4-D 64/240 triethanolamine BR [102]. This compromised the proton gradient across the mitochondrial inner membrane and impaired electron transfer necessary for ATP production during oxidative phosphorylation [133]. Furthermore, various studies in the literature report that the uncoupling of oxidative phosphorylation is a common mechanism of toxicity for chlorophenols [134,135,136]. The mitochondrial respiratory chain represents the major source of intracellular ROS formation, with complexes I and III serving as the major sites of O_2_^−^ production [137,138]. The inhibition of these complexes results in an accumulation of superoxide-generating electron transport intermediates, enhances electron transfer to oxygen, and triggers excessive O_2_^−^ production [137,138]. This contributes to oxidative stress, depletion of antioxidant systems, and damage at cellular and tissue levels [137,138].

Lactate dehydrogenase (LDH) was a frequently disrupted biomarker (*n* = 9, 30%) in articles related to energy metabolism (Figure 4b). LDH participates in energy production through anaerobic metabolism, and the increase in its activity may be associated with low oxygen availability and/or tissue injury [139,140]. Although LDH is not a specific marker of hepatotoxicity, the increase in its levels is related to liver disorders. Rats treated with 126 mg/kg of 2,4-D for approximately 21 days exhibited histopathological damage in the liver and a significant increase in LDH activity [34]. Two days of treatment with 2.5 mg/L of 2,4-D also caused this effect in zebrafish larvae at 120 hpf [4]. Furthermore, different analyzed articles reported that the increase in LDH activity is accompanied by a reduction in antioxidant enzyme activity and hepatic tissue damage [4,32,33,34]. This reinforces the argument that oxidative stress plays a crucial role in the progression of 2,4-D-induced hepatotoxicity. 

#### 3.5.3. Lipid Metabolism

2,4-D also has negative impacts on lipid metabolism, as reported in 18% (*n* = 15) of the reviewed articles. The analyzed biomarkers indicate that 2,4-D induces an increase in fatty acid oxidation in the liver, as evidenced by the elevation in the activity of mitochondrial enzymes involved in the β-oxidation process [37,39,43]. Rats fed a diet containing 0.25% (*w*/*w*) of 2,4-D for seven days showed an increase in the activity of carnitine acetyltransferase (CrAT) and an elevation in the oxidation of palmitoyl-CoA [39]. CrAT catalyzes the reversible transfer of acetyl groups between acyl-coenzyme A and L-carnitine, a fundamental process for transporting short- and medium-chain fatty acids into the mitochondria, where they are oxidized to generate energy [115,141]. Carnitine palmitoyltransferase, fatty acyl-CoA dehydrogenase, and acyl-CoA hydrolase II are also crucial mitochondrial enzymes for fatty acid oxidation, and their activity is increased by herbicide exposure [41,49]. 2,4-D also enhances peroxisomal β-oxidation of fatty acids [42,49]. Peroxisomes are organelles that house vital enzymes for a range of metabolic process, including fatty acid oxidation, phospholipid synthesis, and the maintenance of cellular redox balance [142]. Fatty acid β-oxidation represents a pivotal peroxisomal function, being crucial for shortening the chains of very-long-chain fatty acids that cannot be oxidized in mitochondria [142].

Peroxisomes and mitochondria are significant sources of ROS generation and maintain a close relationship with redox balance [143]. Peroxisomes serve as a major source of H_2_O_2_, which is generated through the activities of various FAD-dependent oxidoreductases involved in peroxisomal metabolic processes, including β-oxidation [144]. Although peroxisomes contain antioxidant enzymes such as catalase, imbalances in H_2_O_2_ levels can compromise antioxidant systems and contribute to oxidative stress [143,144]. Furthermore, disruptions in antioxidant mechanisms and peroxisomal β-oxidation can lead to mitochondrial oxidative stress in different organs, including the liver [144]. This underscores that the increase in fatty acid oxidation may be a significant factor in the generation of oxidative damage induced by exposure to 2,4-D.

#### 3.5.4. Liver Function

Liver markers are used to evaluate liver function and are particularly useful in detecting and monitoring injuries caused by various factors, including toxic chemical compounds. 2,4-D increased the levels of different liver markers, with aspartate aminotransferase (AST) and alanine aminotransferase (ALT) being the most recurrent among the analyzed articles (Figure 4b). The widespread usage of AST and ALT is due to the fact that the AST/ALT ratio is a well-established marker of liver damage [145]. The AST/ALT ratio is relevant because it is a parameter frequently used to assess liver health in both clinical diagnosis and research. Furthermore, its disturbance can indicate different hepatic conditions such as hepatitis, cirrhosis, or hepatic steatosis [118,146]. AST and ALT are enzymes involved in amino acid metabolism, catalyzing the conversion of aspartate and alanine into pyruvate, respectively [146]. Both enzymes are primarily found inside hepatocytes. When liver damage occurs, hepatocyte membranes are compromised, resulting in the release of these enzymes into the bloodstream [145,146]. This leads to an increase in AST and ALT activity and levels in the blood, making them sensitive markers of liver damage [145]. Shafeeq and Mahboob (2021) demonstrated that rats receiving 150 mg/kg/day of 2,4-D for 4 weeks showed increased levels of AST, ALT, and alkaline phosphatase (ALP) [74]. The increase in the enzymatic activity of these three markers was also observed in zebrafish larvae at 120 hpf when treated with 2.5 mg/L of 2,4-D for two days [4].

Alkaline phosphatase (ALP) was the third most mentioned marker of liver function in the analyzed studies. This enzyme is located in the membranes of the biliary canaliculi, structures responsible for bile transport in the liver. An increase in ALP levels indicates dysfunction or obstruction of the biliary flow, a condition that can be caused by liver damage and hepatobiliary disorders [145,147]. Additionally, an increase in total bilirubin levels was also induced by 2,4-D [32,62]. Bilirubin is metabolized by the liver and excreted in the bile, implying that any abnormality in this process can result in its accumulation in the blood [145].

#### 3.5.5. Xenobiotic Metabolism

Exposure to 2,4-D alters the activity of enzymes related to xenobiotic metabolism (Figure 4a). The most frequently occurring markers of this process are GST and GSH. GST plays an important role in the conjugation of xenobiotics with GSH molecules, resulting in the formation of water-soluble conjugates, facilitating their excretion from the body [132]. Additionally, 2,4-D also affects enzymes related to cytochrome P450 (CYP450) [45,57]. CYP450 is a family of enzymes, primarily present in the liver, responsible for the metabolism of a wide range of substances, including xenobiotics [116]. Different isoforms of CYP450 are involved in the metabolism of 2,4-D. Badawi et al. (2000) demonstrated that rats treated with a single dose of 2,4-D (375 mg/L) showed an increase in the expression of CYP1A1, CYP1A2, and CYP1B1 isoforms in the liver [55]. Furthermore, rats receiving doses of 1.6 and 2.9 mg/kg/bw of 2,4-D exhibited changes in the activity of CYP450 and the enzymes ethylmorphine N-demethylase and ethoxyresorufin O-deethylase, which are also part of the cytochrome P450 family [52]. Furthermore, CYP-mediated metabolism can also produce ROS, in addition to bioactivated intermediates, leading to oxidative stress, particularly in the liver, and contributing to liver pathologies [148].

**Figure 5:**
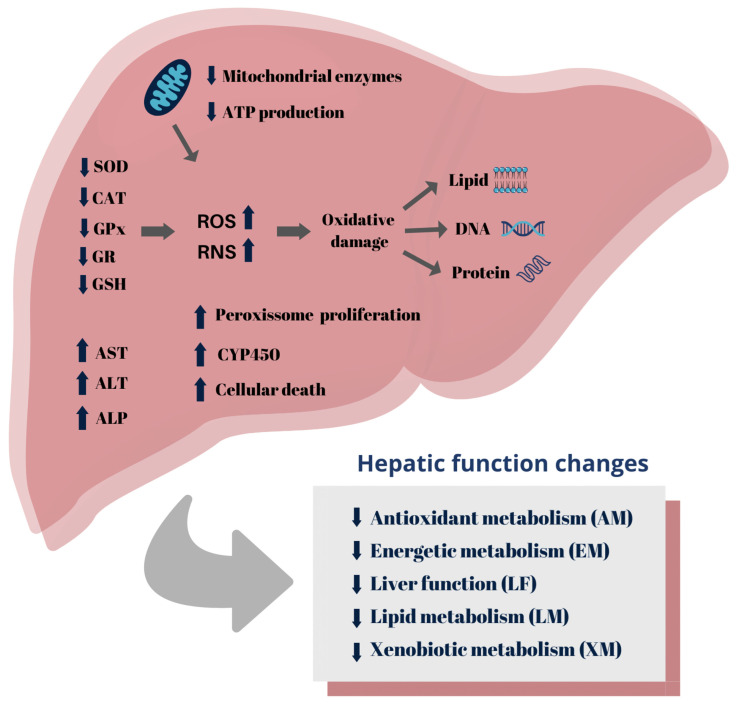
Summary of 2,4-D herbicide induced-hepatotoxicity mechanisms.

Exposure to 2,4-D also induces the proliferation of peroxisomes, organelles that contain a variety of oxidative enzymes important in xenobiotic metabolism [45,57]. Epoxide hydrolases are enzymes, present in peroxisomes, that see increased activity due to exposure to 2,4-D [38,45]. These enzymes function to convert epoxides, intermediates formed during oxidative metabolism by CYP450, into more stable and less reactive metabolites, contributing to the detoxification and elimination of the compound in the body [149]. Figure 5 depicts a schematic representation of the mechanisms underlying the 2,4-D-induced hepatotoxicity reported in Section 3.6 of this study.

### 3.6. Hepatoprotective Assessments

Oxidative stress plays a significant role in the hepatotoxicity induced by 2,4-D [32,33,74]. Therefore, chemical compounds with antioxidant properties are being tested in hepatoprotection assays, aiming to reduce the damage caused by the herbicide (Table 3).

Studies in rats have demonstrated that supplementation with selenium (Se) attenuates the 2,4-D-induced hepatotoxicity. This protection is indicated by the reduction in markers of liver function (AST, ALT, ALP), levels of MDA, and histopathological liver damage, and also by the improvement in the activity of hepatic antioxidant enzymes (CAT, GR, SOD, and GPx) [32,74]. Similar results were also found in rats supplemented with magnesium (Mg) [71]. Se and Mg play essential roles as enzymatic cofactors in antioxidant systems (e.g., for GPx activation and activity), contributing to cell protection against oxidative damage [71,74].

Olive oil and its hydrophilic fraction have also shown promising results against oxidative damage in the livers of 2,4-D-induced rats. The promoted outcomes include the recovery of antioxidant enzyme activity, reduction in the AST/ALT ratio and MDA levels, and the preservation of hepatic histoarchitecture [62,64]. These benefits are attributed to the presence of phenolic compounds (e.g., flavonoids and terpenoids) known for their antioxidant properties, as they have the ability to donate electrons to neutralize free radicals and stimulate antioxidant enzyme activity [33]. The aqueous extract of *Thymus munbyanus*, a plant also rich in phenolic compounds, was also effective against herbicide-induced oxidative damage [33].

### 3.7. Pesticide Mixtures Containing 2,4-D

This review identified studies that evaluated the toxic effects of 2,4-D when combined with other pesticide products. This approach is important as it reflects a more realistic scenario of exposure for non-target organisms. After all, these products are often applied in combination in target crops [150]. Additionally, the mixtures can influence the absorption, distribution, and metabolism of pesticides in non-target organisms, resulting in potential alterations in the toxicity of the individual active ingredients [150,151].

Exposure to commercial formulations of 2,4-D (27 ppm) and azinphosmethyl (0.3 ppm), both individually and in combination for 96 h, produced different results in terms of altering the levels of hepatic antioxidant enzymes in *Oreochromis niloticus* [79]. A synergistic effect between the pesticides was observed regarding SOD activity, while an antagonistic effect was seen in GPx and GR activity [79]. Chaturvedi et al. (1991) tested the effects of 2,4-D alone and in combination with the insecticides toxaphene (TOX) and parathion (PA) in mice and observed different effects on hepatic xenobiotic metabolism enzymes [51]. When administered alone at 50 mg/kg, 2,4-D altered the activities of Amidopyrine N-demethylase and Benzo [a]pyrene hydroxylase. However, when combined with TOX (50 mg/kg) or TOX (50 mg/kg) + PA (5 mg/kg), it induced the activity of other enzymes such as aniline hydroxylase and phenacetin O-dealkylase and increased CYP450 activity [51].

2,4-D was also evaluated in combination with the herbicide picloram, both of which are components of the commercial herbicide formulation Tordon [77]. The mixture of 5.5 mg/L 2,4-D + 0.5 mg/L picloram increased the hepatic ethoxyresorufin 0-deethylase activity in *Ictalurus punctatus* and decreased the liver-to-body weight ratio. These effects were not observed in treatments with individual herbicides, indicating a synergistic effect [77]. These findings highlight the importance of conducting studies that investigate the toxicity of pesticide mixtures given the scarcity of research in this field of research.

### 3.8. Conclusions and Perspectives

In conclusion, 2,4-D has a negative impact on various hepatic biochemical parameters, particularly components of the antioxidant system. This indicates that oxidative damage may play a significant role in the progression of 2,4-D-induced hepatotoxicity. However, despite the advancements made in this field, the mechanism of action, targets, and molecular pathways involved in the herbicide’s hepatotoxicity are not yet fully understood. Comprehending the mechanism of action of herbicides is of paramount importance in the development of more efficient agricultural strategies that minimize risks to the environment and non-target organisms [152,153].

In this context, the use of in silico and in chemico tools has emerged as a viable and efficient alternative for predicting toxicity mechanisms of contaminants (Cotterill et al., 2019). Examples include network analyses (e.g., protein–protein interaction networks) that provide a comprehensive understanding of the interactions between molecular targets and the toxic substance of interest [154,155]. In chemico approaches, such as docking and molecular dynamics, can also be employed to assess the affinity between a chemical compound and different targets, thereby increasing the reliability of the obtained results [156]. Moreover, these approaches make use of toxicological data available in freely accessible databases, such as GeneCards (https://www.genecards.org/, accessed on 30 October 2023) and DisGeNET (https://www.disgenet.org/, accessed on 30 October 2023), and are aligned with the principles of the 3 Rs of animal experimentation (Replacement, Reduction, and Refinement) [157]. Therefore, the use of predictive methodologies in investigating the mechanism of action of 2,4-D offers a promising perspective for advancing our knowledge of its toxicity and contributes to the development of more effective strategies for environmental safety and public health.

## Figures and Tables

**Table 1:** Studies of the hepatotoxicity of 2,4-dichlorophenoxiacetic acid (2,4-D) in in vivo biological models.

Biological Model	Exposure Compounds	Exposure Conditions	Cellular and Tissues Damage	Impaired Biochemical Markers	References
*Chinese Hamsters*	Commercial formulation (550 g/L)	AR: oral gavage T: 9 days C: 100 mg/kg of body weight	NA	ND: Peroxissomes plorifaration	Vainio et al. (1982) [36]
*Rattus novergicus*	Commercial formulation (550 mg/kg)	AR: oral gavage T: 2 weeks C: 100–200 mg/kg of body weight	NA	LM: peroxissome proliferation, CrAT, protein lipases AM: CAT	Vainio et al. (1983) [37]
*Rattus novergicus*	Commercial formulation (550 g/L)	AR.: intragastrically gavage T: 2 weeks C: 100, 150 and 200 mg/kg of body weight	NA	XM: EH, UDPglucuronosyltransferase, GST AM: GST	Hietanen et al. (1983) [38]
*Rattus novergicus*	Active ingredient	AR: feeding T: 14 h C: 0.25% *w*/*w*	NA	LM: CrAT, palmitoyl-CoA, triglycerides AM: CAT	Kawashima et al. (1984) [39]
*Rattus novergicus*	not specified	AR.: feeding T: 14 days C: 0.5% of diet	NA	LM: stearoyl-CoA	Kawashima et al. (1984) [40]
*Rattus novergicus*	Active ingredient	AR: feeding and subcutaneously T: 1 or 2 weeks C: 0.25% of diet or 0.93 mmole or 1.86 mmole per kg of body weight	NA	LM: acyl-CoA hydrolase II; β oxidation	Katoh et al. (1984) [41]
*Rattus novergicus*	Commercial formulation (550 g/L)	AR: intragastrically ET: 14 days CT: 1 mmol/kg of body weight	NA	LM: peroxissome proliferation, β-oxidation AM: GR	Hietanen et al. (1985) [42]
*Mus musculus*	not specified	AR: diet T: 4 days C: ---	Increase liver somatic index	LM: palmitoil-CoA, CrAT EM: cytochrome oxidase	Lundgren et al. (1987) [43]
*Rattus novergicus*	Active ingredient	AR: gavage and feeding T: single dose and 13 days C: 553 mg/kg and 1090 mg/kg (single dose); 0, 15, 60, 100, or 150 mg/kg/day (13 days)	Dose levels of 100 or 150 mg/kg/day produced minimal swelling and increased staining homogeneity in the liver cells and were associated with a slight elevation in liver weight.	LF: ALT, ALP EM: glucose	Gorzinskj et al. (1987) [44]
*Mus musculus*	not specified	AR: feeding T: 4 days C: 100 mg/kg/bw	NA	XM: EH, CYP450, GST, peroxissome proliferation AM: GST	Lundgren et al. (1987) [45]
*Rattus novergicus*	Active ingredient (>99%)	AR.: intragastrically gavage T: 2 weeks C: 100 mg/kg of body weight	NA	XM: peroxisome proliferation, CYP450, UDP-glucunorosyl transferase, NADPH diaphorase	Mustonen et al. (1989) [46]
*Rattus novergicus*	Active ingredient	AR.: feeding T: 7 months C: 0.05% of diet	NA	LM: peroxissome proliferation, acyl Coa oxidase, dicarboxylyl CoA oxidase	Abdellatif et al.. (1990) [47]
*Mus musculus*	Active ingredient (97–99%)	AR.: oral intubathion T: 14 days exposure + 7 days recovery C: 50 mg/kg	Increase liver/body weight ratio	LF: ALT	Kuntz et al. (1990) [48]
*Rattus novergicus*	Active ingredient	AR: feeding T: 6 days C: 1.680 ppm	NA	LM: CrAT; carnitine palmitoyltransferase fatty acyl-CoA dehydrogenase cyanide-insensitive fatty acyl-CoA, peroxissome proliferation AM: CAT	Kozuka (1991) [49]
*Rattus novergicus*	not specified	AR.: oral T: 2 weeks C: 200 mg/kg/day	NA	XM: NADPH cytocrome C reductase, aniline hydroxylase, Cytocrome B, NADPH ferricicyanide reductase, aminopyrine N-demethylase	N Inomata et al. (1991) [50]
*Mus musculus*	Active ingredient (>97%)	AR: oral intubation T: 7 days C: 50 mg/kg of body weight	NA	XM: amidopyrine N-demethylas, Benzo [a]pyrene hydroxilase	Chaturvedi et al. (1991) [51]
*Rattus novergicus*	Commercial formulation	AR: oral and middorsal skin applications T: single dose C: 1.9 and 2.6 mg/kg of body weight	NA	XM: CYP450, ethylmorphine N-demethylase, ethoxyresorufin O-deethylase	Knopp and Schiller (1992) [52]
*Rattus novergicus*	not specified	AR: oral T: single dose; 30 days and 180 days C: 600 mg/kg (single dose) and 200 ppm (30 and 180 days)	NA	LF: AST, ALT, ALP EM: LDH, amylase, glucose ND: creatinine	Paulino et al. (1996) [53]
*Mus musculus*	Active ingredient	AR.: feeding T: 4 days C: 0.125% of diet	NA	LF: mdr2 gene	Miranda et al. (1997) [54]
*Rattus novergicus*	Active ingredient	AR: oral gavage T: single dose C: 375 mg/L	NA	XM: CYP1A1, CYP1A2, CYP1B1	Badawi et al. (2000) [55]
*Rattus novergicus*	Active ingredient (>98%)	AR: injections T: 30 days C: 70 mg/kg of body weight	NA	EM: mitochondrial dysfunction	Di Paolo et al. (2001) [56]
*Rattus novergicus*; *Mus musculus* and Syrian hamsters	Active ingredient	AR: feeding T: 3 months C: 0, 12, 28, 83, 250, 700, and 1680 ppm (*M. musculus*); 0, 17, 83, 250, 750, 1250, and 2500 ppm (*R. novergicus*); 0, 12, 100, 500, 1000, and 5000 ppm (*Syrian hamsters*)	Increase of mice liver weith	XM: CYP450; peroxissome proliferation AM: CAT	Ozaki et al. (2001) [57]
*Mus musculus*	Active ingredient	AR: feeding T: 6 days C: 1.680 ppm	NA	ND: c-myc gene	Ge et al. (2002) [58]
*Mus musculus*	Active ingredient	AR: Intraperitoneally T: 55 days C: 3.8 mg/kg bw	NA	EM: LDH, MDH	Yilmaz and Yuksel (2005) [59]
*Rattus novergicus*	not specified	AR: drink water T: 25 days C: 50 and 100 ppm	NA	AM: SOD, GSH, GR, MDA EM: LDH, creatine kinase LF: AST XM: GSH	Celik et al. (2006) [60]
*Rattus novergicus*	Active ingredient	AR: Feed and drink water T: 30 days C: 25 ppm and 50 ppm (water) and 50 ppm and 100 ppm (food)	No hepatic damage was observed, but the level of 2,4-D in the liver was found to be significantly higher in both the feed and water groups compared to the control group.	NA	Aydin et al. (2006) [61]
*Rattus novergicus*	Active ingredient	AR: drink water T: 21 days C: 600 ppm or 126 mg/kg	Vascular congestion, cytoplasmic vacuolization, and mononuclear cells’ infiltration	AM: SOD, CAT, GPx, MDA LF: AST, ALT, ALP, γ-glutamyl transpeptidase EM: LDH	Troudi et al. (2012) [34]
*Rattus novergicus*	Commercial formulation (600 g/L)	AR: oral gavage T: 4 weeks C: 5 mg/kg/bw	NA	AM: SOD, CAT, GPx, GR, MDA LF: AST, ALT, ALP, γ-GGT, total bilirubin. LM: change in fatty acid composition	Nakbi. et al. (2010) [62]
*Rattus novergicus*	Commercial formulation (600 g/L)	AR: oral gavage T: 4 weeks C: 15, 75 and 150 mg/kg of body weight	Body weight decreased and the liver weight increased significantly. 2,4-D induced hepatic cord disruption, focal necrosis, vessel dilation and pycnotic nucleus.	LF: AST, ALT, ALP, γ-GGT AM: CAT, GR	Tayeb et al. (2010) [63]
*Rattus novergicus*	Commercial formulation (600 g/L)	AR: oral gavage T: 4 weeks C: 5 mg/kg of body weight/day	Vascular congestion and wide sinusoidal spaces and a necrotic	AM: SOD, CAT, GPx, MDA LF: AST, ALT LM: low-density lipoprotein, cholesterol	Nakbi et al. (2012) [64]
*Rattus novergicus*	Commercial formulation (600 g/L)	AR: oral gavage T: 28 days C: 15, 75 and 150 mg/kg/bw/day	NA	AM: SOD, CAT, GPx, GR, MDA LM: change in fatty acid composition	Tayeb et al. (2013) [65]
*Rattus novergicus*	not specified	AR: feeding T: 16 weeks C: 200 mg/kg/day	2,4-D acid iso-octylester caused the formation of atypical cell foci (ACF) in the pancreata and livers of rats.	NA	Kalipici et al. (2013) [66]
*Rattus novergicus*	Active ingredient (≥90%)	AR: oral gavage T: 19 days C: 100 mg/kg of body weight	NA	AM: CAT, MDA, total antioxidant capacity	Mazhar. et al. (2014) [67]
*Rattus novergicus*	Commercial formulation	AR: oral T: 28 days C: 75 or 150 mg/kg of body weight	2,4-D increased liver weight and induced nuclear changes in liver cells, including alterations in size and shape, irregularity, and slight distention of nuclear envelope, hepatic nuclei exhibited varying degrees of pyknosis, disaggregation and apoptosis.	LF: AST, ALT, ALP, total bilirubin AM: GR, SOD EM: LDH	Al-Baroudi et al. (2014) [68]
*Rattus novergicus*	Commercial formulation	AR: oral gavage T: 24 h (single dose) C: 639 mg/kg of body weight	NA	AM: hydroperoxyl and carbonyl lipids EM: glycogen	Dakhakhni et al. (2016) [69]
*Mus musculus*	Active ingredient	AR: oral T: 45 days C: 30, 60, 90 mg/kg/day	Vascular and hepatocellular lesions with necrotic changes and focal areas of necrosis in the liver.	AM: GSH, SOD, CAT, GPx, GR, GST and total –SH EM: ATP and SDH XM: GSH and GST	Satapathy and Rao (2018) [70]
*Rattus novergicus*	Active ingredient	AR: oral gavage T: 4 weeks C: 150 mg/kg/day	NA	AM: SOD, CAT, GSH, MDA LF: AST, ALT XM: GSH ND: Urea and creatinine	Shafeeq and Mahboob (2020) [71]
*Rattus novergicus*	Commercial formulation (806 g/L)	AR: inhalation and feed T: 6 months C: 3.71/6.19 and 9.28×10^−3^ g a.i./ha	The groups exposed to oral 2,4-D had a higher incidence of steatosis, and those exposed to high doses had increased liver inflammation.	LF: ALT	Bonfim et al. (2020) [72]
*Rattus novergicus*	Commercial formulation (600 g/L)	AR: oral gavage T: 4 weeks C: 5 mg/kg/bw/day	Rat livers shown perivascular inflammatory infiltration around the vessel, sinusoidal dilatation and vacuolization of hepatocytes.	AM: SOD, CAT, GSH, GPx, GST, MDA LF: AST, ALT, ALP, total bilirubin EM: LDH XM: GST, GSH	Tichati et al. (2020) [32]
*Rattus novergicus*	Active ingredient (>98%)	AR: cannulation of portal and cava veins liver T: 20 min. C: 10–400 µM	Membrane lipid bilayer deformity	EM: NADH, NAD^+^, lactate, glycolisis, gluconeogenesis	Salla et al. (2019) [73]
*Rattus novergicus*	Active ingredient	AR: oral gavage T: 4 weeks C: 150 mg/kg/day	NA	AM: SOD, CAT, GSH, MDA LF: AST, ALT, ALP XM: GSH ND: urea and creatinine	Shafeeq and Mahboob (2021) [74]
*Rattus novergicus*	Commercial formulation (600 g/L)	AR: oral gavage T: 30 days C: 5 mg/kg/bw	2,4-D increases relative and absolute liver weights. Furthermore, 2,4-D induces severe infiltration of mononuclear inflammatory cells with vacuolar degeneration around a dilated central lobular vein, congestion of the hepatic sinusoids, and degenerative hepatocytes with largely vacuolated cytoplasm and a large number of lipid droplets.	AM: SOD, CAT, GPx, GST, MDA, carbonyl proteins LF: AST, ALT, ALP, γ-GGT EM: LDH XM: GST, GSH	Tichati et al. (2021) [33]
*Rattus novergicus*	Commercial formulation (480 g/L)	AR: oral T: 60 days C: 5 mg/kg of body weight	In the liver tissue of rats, focal areas of mononuclear cell infiltration in the pericentral and periacinal region, sinusoidal dilatation, and hyperemia in the vessels and areas of pyknosis and parenchymal degeneration in the nuclei of hepatocytes were determined.	LF: AST, ALT, ALP AM: SOD, GSH, CAT, MDA XM: GSH ND: NF-κB, COX-2, TNF-α, MCP-I, TGFβI, and CYP2E P53, Bax/Bcl-2, caspase-3, caspase-8, caspase-9, and PARP	Sinan Ince et al. (2022) [75]
*Rattus novergicus*	2,4-D, gliphosate and dicamba (not specified)	AR: drink water T: 90 days C: gliphosate (0.5 mg/kg bw/day) + 2,4-D (0.3 mg/kg bw/day) + dicamba (0.02 mg/kg bw/day)	NA	AM: GSH and MDA	Nechalioti et al. (2023) [76]
*Ictalurus punctatus*	Active ingredient 2,4-D (>99%) Picloram (>99%)	AR: water expossure T: 10 days C: 22.5, 7.5, and 2.25 mg/L	NA	XM: ethoxyresorufin 0-deethylase	Gallagher and Digiulio (1991) [77]
*Cyprinus carpio*	Active ingredient (>98%)	AR: water exposure T: 96 h and 14 days C: 310, 295 and 270 mg/L (96 h) 150, 200, and 250 mg/L (14 days)	Hepatocycites shown slight vacuolar degeneration and pycnotic nuclei (some of them displaced).	LF: AST, ALT	Neskovic et al. (1994) [78]
*Oreochromis niloticus*	Commercial formulation (500 g/L)	AR: water exposure T: 96 h C: 27 ppm	NA	AM: SOD, GPx, GR EM: *glucose-6-phosphate dehydrogenase*	Oruç. and Uner (2000) [79]
*Fundulus heteroclitus*	not specified	AR: water expossure T: 21 days C: 0.04, 0.41, and 4.1 µM	NA	ND: peroxissome proliferation	Ackers et al. (2000) [80]
*Cyprinus carpio*	Commercial formulation (500 g/L)	AR: water exposure T: 96 h C: 87 ppm	NA	AM: GST, SOD EM: G6PD XM: GST	Oruç and Uner (2002) [81]
*Leporinus obtusidens*	Commercial formulation (868 g/L)	AR: water exposure T: 96 h C: 1 and 10 mg/L	NA	EM: glycogen, lactate, glucose	Fonseca et al. (2008) [82]
*Rhamdia quelen*	Commercial formulation (720 g/L)	AR: water exposure T: 96 h C: 0, 400, 600 and 700 mg/L	Hepatocyte vacualization and changes in its arrangement cords.	EM: glycogen, lactate, glucose	Cattaneo et al. (2008) [83]
*Carassius auratus*	Active ingredient	AR: water exposure T: 90 h C: 1, 10 and 100 mg/L	NA	AM: carbonyl proteins, lipid peroxidases LM: lipid peroxidases	Matviishyn et al. (2014) [84]
*Poecilia vivipara*	Commercial formulation (868 g/L)	AR: water exposure T: 48 h C: 10, 20 and 40 μL	Swollen nuclei and cytoplasmic vacuolization. Finally, the 40 μL/L group presented blood vessel alterations indicating vasodilatation, hepatocytes with swollen nuclei, Ito cells, and micronuclei.	NA	Vigário and Sabóia-Morais (2014) [85]
*Rhamdia quelen*	Commercial formulation (720 g/L)	AR: water exposure T: 90 days C: 0.5 and 2 mg/L	NA	AM: CAT, MDA EM: glycogen; lactate, glucose	Menezes. et al. (2015) [86]
*Cyprinus carpio* L	not specified	A.R.: water exposure T: --- C: 0.2 mg/dm^3^	NA	EM: ICDH, LDH, G6PD	Yakovenko et al. (2018) [87]
*Capoeta capoeta*	not specified	AR: water expossure T: 7 days C: 10 and 20 mg/L	NA	AM: plasma oxidative status index LF: AST	Kaya et al. (2018) [88]
*Danio rerio*	Active ingredient (>97%)	AR: water exposure T: 48 h C: 2.5, 5 and 10 mg/L	Hepatocytes had heterogeneous eosinophilic, cytosol vacuolization and cell nucleus were eccentric. Loss of cell boundaries and liver with necrotic appearance. Release of cytosolic content among adjacent cells.	LF: AST, ALT, ALP AM: CAT, GST XM: GST EM: LDH	Martins et al. (2021) [4]
*Triturus cristatus carnifex*	Commercial formulation (37% of 2,4-D as iso-octylic ester)	AR: water exposure T: 3 months C: 25, 50, 75, 100, 125, and 150 ppm	Vacuolar degeneration of liver parenchyma and necrosis of kidney tubules.	NA	Zaffaroni et al. (1986) [89]
*Lithobates clamitans*	Active ingredient (>98%)	AR: soil exposition T: 2 days C: 14.3 µg/cm^2^	NA	NA	Van Meter et al. (2018) [90]
*Physalaemus albonotatus*	Commercial formulation) (48.5% *w*/*v* of active ingredient)	AR: water exposure T: 96 h (acute) and 49 days (chronic) C: 350, 700, 1400, and 2400 mg/L(acute); 43.7, 87.5, 175 or 262.5 mg/L (chronic)	The liver of treated tadpoles showed enlargement of hepatic sinusoids, hypervascularization, dilation of blood vessels, and vacuolization of hepatocytes	NA	Curi et al. (2019) [91]

Abbreviations: (AR) administration route; (T) time; (C) concentrations; (AM) antioxidant metabolism; (EM) energetic metabolism; (LF) liver function; (LM) lipid metabolism; (XM) xenobiotic metabolism; (ND) Not determined; (NA) no analyzed; (NS) not specified; (---) information not informed by the authors. Abbreviations: (AR) administration route; (T) time; (C) concentrations; (AM) antioxidant metabolism; (EM) energetic metabolism; (LF) liver function; (LM) lipid metabolism; (XM) xenobiotic metabolism; (ND) not determined; (NA) not analyzed; (NS) not specified; (CAT) catalase; (GST) glutathione S-transferase; (GPx) glutathione peroxidase; (CrAT) carnitine acetyltransferase; (EH) epoxide hydrolases; (GR) glutathione reductase; (ALT) alanine aminotransferase; (AST) aspartate aminotransferase; (ALP) alcaline phosphatase; (CYP450) cytochrome P450; (LDH) lactate dehydrogenase; (MDH) malate dehydrogenase; (SOD) superoxide dismutase; (GSH) reduced glutathione; (MDA) malondialdehyde; (γ-GGT) gamma-glutamyltransferase; (SDH) succinate dehydrogenase; (G6PD) glicose-6-fosfato desidrogenase; (IDH) isocitrate dehydrogenase.

**Table 2:** Studies of the hepatotoxicity of 2,4-dichlorophenoxiacetic acid (2,4-D) in in vitro biological models.

Biological Model	Exposure Compounds	Exposure Conditions	Impaired Biochemical Markers	References
Liver GST of *Rattus novergicus*	Active ingredient	AR.: enzyme kinetics T: --- C:---	AM: GST XM: GST	Dierickx (1983) [92]
Liver GST of *Rattus novergicus*	Active ingredient (>99%)	AR: enzyme kinetics T: --- C: 2–12 mM	XM: GST AM: GST	Vessey and Boyer (1984) [93]
Liver GST of *Salmo gairdneri*	Active ingredient	AR: enzyme kinetics T: --- C: 2 mM	AM: GST XM: GST	Dierick (1985) [94]
Liver GST of *Homo sapiens* (autopsy)	Active ingredient (>97%)	AR: --- T: --- C: ---	AM: GST XM: GST	Singh (1985) [95]
Liver GST of *Cyprinus carpio*	not specified	AR: cell culture T: --- C: ---	AM: GST XM: GST	Elia et al. (2000) [96]
Liver GST of *Rattus novergicus*	not specified	AR: --- T: --- C: ---	AM: GST XM: GST	Dierickx (1988) [97]
Liver GST of *Chalcalburnus tarichii Pallas*	Active ingredient	AR.: --- T: --- C: 0.6, 0.23 and 0.57 mM	AM: GST XM: GST	Özaslan et al. (2018) [98]
Liver mitochondria of *Rattus novergicus*	Active ingredient	AR.: cell culture T: --- C: 0, 0.2, 0.5, 1.0, and 2 mM.	LM: palmitoyl CoA hydrolase, fatty acyl CoA EM: mitochondrial dysfunction	Dixon et al. (1990) [99]
Liver mitochondria of *Rattus novergicus*	not specified	AR: cell culture T: --- C: 0.1–4.0 mM	EM: mitochondrial dysfunction	Zychlinski and Zolnierowicz (1990) [100]
Liver mitochondria of *Rattus novergicus*	Active ingredient	AR: cell culture T: --- C: 100, 200, 300, 400, 500, 600, 700 and 800 µM	EM: SDH, cytochrome c reductase, mitochondrial dysfunction	Palmeira et al. (1994) [101]
Liver mitochondria of *Rattus novergicus*	Commercial formulation (2,4-D 1.08 M + Picloram 0.265 M)	AR: cell culture T: --- C: 66.2 nmol picloram + 270 nmol 2,4-D mg^−1^ protein	EM: NADH oxidase, NADH cytochrome c reductase, ATP, mitochondrial dysfunction	Pereira et al. (1994) [102]
Liver mitochondria of *Rattus novergicus*	Commercial formulation. Tordon (2,4-D 300 g/L + picloram 75 g/L)	AR.: cell culture T: --- C: ---	EM: mitochondrial dysfunction	Oakes and Pollak (1999) [103]
Liver *Rattus novergicus* mitochondria	Active ingredient (>98%)	AR: injections T: 30 days C: 70 mg/kg of body weight	EM: mitochondrial dysfunction	Di Paolo et al. (2001) [56]
Hepatocytes of *Rattus novergicus*	Active ingredient	AR: cell culture T: --- C: 1–10 mM	EM: LDH, ATP, ADP, AMP, NADH, NAD^+^ AM: GSH, GSSG XM: GSH, GSSG	Palmeira et al. (1994) [104]
Hepatocytes of *Rattus novergicus*	Active ingredient (>98%)	AR: cell culture T: 200 min C: 1, 5 and 10 mM	AM: MDA, proteins thiol, GSH XM: GSH	Palmeira et al. (1995) [105]
Hepatocytes of *Rattus novergicus*	Active ingredient	AR: cell culture ET: 3 months C.T: 1 mM	NA	Li et al. (2003) [106]
Hepatocytes of *Metynnis roosevelti*	Active ingredient	AR: cell culture T: --- C: 0.275, 2.75 and 27.5 mg/L	EM: mitochondrial dysfunction	Salvo et al. (2015) [107]
HepG2 cells	Active ingredient	AR: cell culture T: 48 h C: 4, 8 and 16 mM	EM: mitochondrial dysfunction ND: Cell cicle alterations, apoptose, DNA damage	Tuschl and Schwab (2003) [108]
HepG2 cells	Active ingredient	AR: cell culture T: 48 h C: 8, 14 and 16 mM	ND: Cell cycle alterations, apoptosis, DNA damage	Tuschl and Schwab (2004) [109]
HepG2 cells	Commercial formulation	AR: cell culture T: --- C: 0.1 nM to 4 mM	ND: Genes involved in stress response, cell cycle control, immunological and DNA repair genes. (FTH1, FTL, PCNA, DCLRE1C, TCLK1, JM11, VEGF, USP19, DDB2, IL1RL1, PTGER3 and GTF2A.)	Bharadwaj et al. (2005) [110]
HepG2 cell	Active ingredient (>90%)	AR.: cell culture T: --- C: 0.001–0.1 mM	NA	Barrón Cuenca et al. (2022) [111]
Liver homogenates of *Rattus novergicus*	Active ingredient	AR: cell culture T: --- C: ---	LM: cholesterol	Olson et al. (1974) [112]
Chicken embryo	Commercial formulation (37%)	AR.: injected into the air cell of the eggs T: 19 days C: 1, 2 and 4 mg/egg	XM: ethoxycoumarin O-deethylase, GST AM: GST	Santagostino et al. (1991) [113]
Chicken Liver	Commercial formulation (31.6% *w*/*v*)	AR: fertilized eggs were externally treated T: 21 days C: 3.1 mg	EM: G6Pase LM: total lipids AM: CAT	Duffard et al. (1993) [114]

Abbreviations: (AR) administration route; (T) time; (C) concentrations; (AM) antioxidant metabolism; (EM) energetic metabolism; (LF) liver function; (LM) lipid metabolism; (XM) xenobiotic metabolism; (ND) not determined; (NA) not analyzed; (NS) not specified; (---) information not informed by the authors. Abbreviations: (AR) administration route; (T) time; (C) concentrations; (AM) antioxidant metabolism; (EM) energetic metabolism; (LF) liver function; (LM) lipid metabolism; (XM) xenobiotic metabolism; (ND) not determined; (NA) not analyzed; (NS) not specified; (CAT) catalase; (GST) glutathione S-transferase; (G6Pase) glucose 6-phosphatase; (LDH) lactate dehydrogenase; (GSH) reduced glutathione; (MDA) malondialdehyde; (SDH) succinate dehydrogenase.

**Table 3:** Hepatoprotection studies against 2,4-D herbicide induced-hepatotoxicity.

Biological Model	Hepatoprotective Agent	Concentrations and Time of Exposure	Hepatoprotective Effects	References
*Rattus novergicus*	Extra virgin olive oil (EVOO) and its hydrophilic fraction (OOHF)	C: 2,4-D (5 mg/kg body weight) + EVOO (300 μL/day) or OOHF (1 mL/day) T: 4 weeks	EVOO and OOHF supplementation induced a significant increase in the antioxidant enzyme activities (SOD, CAT, GPx and GR) and liver markers (AST, ALT and total bilirubin) and a decrease in the conjugated diene (CD) and thiobarbituric acid-reactive substance (TBAR) levels in the liver.	Nakbi, A. et al. (2010) [62]
*Rattus novergicus*	Extra virgin olive oil (EVOO) and its hydrophilic fraction (OOHF)	C: 2,4-D (5 mg/kg body weight) + EVOO (300 μL/day) or OOHF (1 mL/day) T: 4 weeks	EVOO and OOHF supplementation induced a significant increase in the antioxidant enzyme activities (SOD, CAT, GPx) and liver markers (AST, ALT and total bilirubin), and decreased MDA levels in the liver.	Nakbi, A. et al. (2012) [64]
*Rattus novergicus*	Chamomile capitula extract	C: 2,4-D (75 or 150 mg/kg body weight) + Chamomile capitula extract—(500 mg/kg body weight) T: 28 days	Chamomile capitula extract presented antioxidant effects, improving the levels of SOD and GR. The levels of hepatic enzymes AST, ALT, ALP, and LDH decreased, as well as levels of total bilirubin. Additionally, the degenerative damages in the hepatic tissue caused by 2,4-D were also alleviated.	Al-Baroudi et al. (2014) [68]
*Mus musculus*	Curcumin	C: 2,4-D (30, 60, 90 mg/kg/day) + Curcumin (10 mg/kg/day) T: 45 days	Curcumin supplementation exhibited antioxidant effects, mainly normalizing the levels of GSH, GR, and lipid peroxidation. Furthermore, curcumin supplementation reduced hepatic tissue damage.	Satapathy and Rao (2018) [70]
*Rattus novergicus*	Magnesium (Mg)	C: 2,4-D (150 mg/kg body weight/day) + Mg supplement (50 mg/kg body weight/day) T: 4 weeks	Mg supplementation exhibited its antioxidant properties by significantly improving urea, creatinine SOD, MDA, CAT, GSH and MDA levels and antioxidant enzyme activities. Hepatic markers were also improved: AST, ALT and ALP and absolute liver weight.	Shafeeq and Mahboob (2020) [71]
*Rattus novergicus*	Selenium (Se)	C: 2,4-D (5 mg/kg body weight/day) + Se supplement (1 mg/kg body weight/day) T: 4 weeks	Se supplementation in 2,4-D-treated rats elicited a reduction in the toxic effects of the pesticide by improving the studied parameters (absolute liver weight, total bilirubin, AST, ALP, LDH, MDA and carbonyl proteins), which was confirmed by the histological study of the liver.	Tichati, L. et al. (2020) [32]
*Rattus novergicus*	Selenium (Se)	C: 2,4-D (150 mg/kg body weight/day) + Se supplement (1 mg/kg body weight/day) T: 4 weeks	Se supplementation exhibited its antioxidant properties by significantly improving urea, creatinine, ALP, AST, and ALT, and MDA levels and antioxidant enzyme activities. Hepatic and renal toxicities were attenuated via Se supplementation.	Shafeeq and Mahboob (2021) [74]
*Rattus novergicus*	*Thymus munbyanus* extract (AETM)	C: 2,4-D (5 mg/kg body weight) + AETM (10 mL/kg body weight) T: 30 days	AETM supplementation showed a marked enhancement in the above altered hepatic functional and antioxidant parameters (CAT, GST, total bilirubin, AST, ALP, MDA, carbonyl proteins) and liver histopathology.	Tichati, L. et al. (2021) [33]

Abbreviations. (C): concentration, (T): time of exposure.

## Data Availability

Not applicable.
